# Cognitive Behavioral Digital Interventions are Effective in Reducing Anxiety in Children and Adolescents: A Systematic Review and Meta-analysis

**DOI:** 10.1007/s10935-023-00760-0

**Published:** 2023-12-14

**Authors:** Luca Csirmaz, Tamás Nagy, Fanni Vikor, Krisztian Kasos

**Affiliations:** 1 Doctoral School of Psychology, ELTE Eötvös Loránd University, Budapest, Hungary; 2 MTA-ELTE Lendület Adaptation Research Group, Institute of Psychology, ELTE Eötvös Loránd University, Budapest, Hungary; 3 Institute of Psychology, ELTE Eötvös Loránd University, Izabella u. 6, Budapest, 1064 Hungary

**Keywords:** Anxiety, Intervention, Children, Digital, Technology-mediated, Meta-analysis

## Abstract

Anxiety disorders are the most prevalent mental health issues in childhood, yet most children do not receive treatment. With recent advances in technology, a growing number of digital anxiety interventions are becoming accessible. This study is the first meta-analysis to evaluate the effectiveness and usefulness of digital cognitive-behavioral anxiety interventions for individuals under the age of 18. Five electronic databases (PsycNET, Web of Science, Science Direct, Pub Med, SAGE Journals.) were systematically searched in 2021. Inclusion criteria were: randomized control trials with a wait list no treatment control, standalone to blended care with minimal therapist involvement, diagnosed anxiety disorder or elevated levels of anxiety, outcome anxiety levels had to be assessed by a clinician, or the patients themselves by a validated anxiety measure. We assessed and controlled for publication bias, and considered the risk of bias using the Cochrane risk-of-bias tool. Eighteen studies were found eligible, which examined 1290 participants in total. Pooled effect sizes using a random-effects model yielded low overall effect for self-ratings (g = 0.28, k = 18, *p* < 0.001, 95% CI [0.14; 0.41]), and medium effect for ratings of clinicians (g = 0.66, k = 13, *p* < 0.001, 95% CI [0.50; 0.80]) as well as for parental report (g = 0.49, k = 16, *p* < 0.001, 95% CI [0.29; 0.69]). We found that the effects were homogenous across studies. Further examining potential moderating factors of treatment efficacy is needed in future research, as well as conducting studies that compare traditional methods of care to their digital counterparts. Digital interventions could contribute to the well-being of children regardless of age, minimizing therapist involvement. We conclude that digital cognitive-behavioral interventions may provide an accessible, cost-effective, and scalable anxiety treatment option for children and adolescents.

**Supplementary Information:**

The online version contains supplementary material available at 10.1007/s10935-023-00760-0.

## Introduction

Anxiety disorders represent the most pervasive psychiatric conditions in childhood, afflicting up to 20% of the pediatric population (American Psychiatric Association, 2013; Bandelow & Michaelis, 2015). These disorders are linked to multifaceted detrimental consequences, including impaired self-esteem, compromised social and familial relationships, hindered academic performance, and heightened risk for substance abuse (Brendel, 2011). Research underscores the significance of early-onset anxiety disorders, as they are associated with exacerbated mental health complications in later life, such as comorbid generalized anxiety disorder or major depressive disorder (Ramsawh et al., 2011). Left unaddressed, anxiety disorders may precipitate severe adult challenges, encompassing suicidal ideation, suicide attempts (Brent et al., 1986), and addiction to alcohol or drugs (Benjamin et al., 2013).

Psychological treatments, including mindfulness (Zoogman et al., 2015) and cognitive behavioral therapy (CBT; Pahl & Barrett, 2010), have demonstrated efficacy in ameliorating anxiety symptoms in various age groups (Cartwright-Hatton et al., 2004; Smits et al., 2008). Meta-analytic evidence reveals that approximately 70% of childhood anxiety disorder patients achieve remission following CBT, no longer meeting primary diagnostic criteria (In-Albon & Schneider, 2006). Furthermore, a recent meta-analysis supports CBT's role in enhancing post-treatment remission rates (James et al., 2020).

Multiple barriers, such as location, time, cost (Gunter & Whittal, 2010), and stigmatization (Kaushik, 2016), can hinder access to psychotherapy for children and their families. The COVID-19 pandemic has exacerbated these challenges, with heightened service demands and extensive waiting lists obstructing traditional help-seeking avenues (Radez et al., 2021).

Fortunately, recent technological advancements have facilitated the development of location-independent digital interventions, allowing clients to partially or fully control their treatment (Hall & Bierman, 2015; MacDonell & Prinz, 2017; Miller et al., 2010; Yap et al., 2018). Furthermore, meta-analyses have demonstrated the effectiveness of computer-based interventions in reducing anxiety and depression in adults (Andrews et al., 2010, 2018). Moreover, for minors, these programs are delivered through parents or technology, often incorporating limited therapist involvement via phone calls or emails. Notably, internet-based cognitive behavioral therapy (iCBT) has been shown to produce effects equivalent to traditional CBT in adults (Hedman et al., 2012). Several reviews and meta-analyses have explored the efficacy of technology-delivered interventions for children and adolescents. Vigerland et al. (2016a, 2016b) found moderate effect sizes for internet-delivered CBT (ICBT) compared to waitlist controls, suggesting successful adaptation to a digital format. Similarly, Podina et al. (2016) reported that technology-mediated CBT is as effective as standard CBT in reducing youth anxiety symptoms. However, Donovan and March (2014) expressed concerns about intervention heterogeneity, with some interventions being more promising than others in reducing certain disorders. Girst et al. (2019) conducted a comprehensive meta-analysis comparing various technology-delivered interventions, revealing small effect sizes in favor of digital interventions and medium effect sizes for CBT interventions. The significance of parental and therapist support was observed, but not separately examined for different treatments, highlighting the need for careful comparison to develop effective interventions.

Smartphone based interventions are new, but promising youth-directed interventions for anxiety reduction (Cumino et al., 2017; Stoll et al., 2017). Other youth-directed — but maybe less future-proof — methods include CD-ROMs such as The Cool Teens (Wuthrich et al., 2012) or self-directed cognitive behavioral programs like MoodGYM (Calear et al., 2009). MacDonell and Prinz (2017) reviewed technology-based interventions focused either on youth or family. They found that youth-focused interventions showed some promise in reducing anxiety/depressive symptoms as well as family-focused interventions addressing anxiety symptoms and bolstering parenting efficacy.

In order to understand underlying variables of treatment efficacy, potential moderators should be examined. There is evidence suggesting parental factors to be especially important moderators of treatment efficacy (Yap et al., 2018). Therefore, based on this important moderator of treatment efficacy the two main directions of digital interventions for children can be separated into youth-focused, and family-focused programs (MacDonell & Prinz, 2017). These interventions differ in whether the intervention is directed only at children, or also involve active engagement of parents. There is evidence that points to the value of involving parents across the mental health continuum (Yap et al., 2018). Moreover, evidence suggests a genetic and environmental intergenerational transmission of anxiety (Brendel, 2011). Thus, it would be important to examine whether these interventions prove to be more efficacious than the solely youth-focused ones. For example there is a fully automated web-based parenting intervention which showed some effects on parenting behaviors that are associated with adolescent’s risk for depression and anxiety (Yap et al., 2018). Esbjørn and Walczak (2016) developed and tested a parent-based self-help program with minimal therapist involvement, providing a cost-effective way of treatment for children with moderate anxiety.

In this meta-analysis of randomized controlled trials, we assessed the effectiveness of digital CBT treatments for anxiety in young individuals, considering both technology-delivered and parent-assisted interventions. Our study contributes to the existing literature by focusing on a continuum of family-focused and youth-focused CBT interventions, offering unique insights by comparing different treatment outcomes, such as parental and clinician ratings, instead of relying solely on self-report (e.g., Girst et al., 2019). Additionally, we examined underlying factors potentially influencing treatment efficacy, including therapist involvement, age, gender, intervention length, and study quality.

We hypothesize that (1) digital interventions are efficient in reducing anxiety levels amongst children and adolescents, and that (2) family-focused interventions are more effective in anxiety reduction and prevention than youth-directed ones (based on Brendel, 2011; MacDonell & Prinz, 2017; Yap et al., 2018). We explore the effects of different moderator variables that may impact treatment efficacy.

## Method

### Preregistration and Open Science Practices

This meta-analysis protocol was preregistered on December 2, 2019 and is publicly accessible at the following link: https://osf.io/7su4g. The preregistration was carried out before data collection and preceded any statistical analysis. All data and analysis code are available at the project’s OSF page (see Appendix B).

### Inclusion/Exclusion Criteria

For studies to be included in the meta-analysis, the following criteria had to be met: (1) the study had to be a randomized controlled trial. (2) Participants in the study had to have a diagnosed anxiety disorder or elevated symptoms of anxiety compared to the ‘normal range’ of the specific measure. (3) Anxiety disorders or levels had to be either diagnosed by a clinician, or assessed by a research team by diagnostic interview or cut off scores on a validated anxiety measure. (4) Outcome anxiety levels had to be assessed by either a clinician, or a validated anxiety measure. (5) All participants had to be below 18 years old. (6) Intervention had to be primarily delivered at home, or through an electronic device (computer, phone). (7) Intervention had to be based on cognitive behavioral methods (based on whether the term cognitive behavioral is used in an article’s methods section). (8) Intervention had to be either standalone, unguided, guided or blended care with minimal therapist involvement (9) Also, studies where control groups started or received any other form of treatment were not involved (with the exception of allowing participants to continue their ongoing medication). (10) Studies in which only the parents received training from a therapist were also excluded. (11) We did not exclude studies based on the intervention length. (12) Only published journal articles were included.

### Search Strategy

Five electronic databases were systematically searched for studies including: PsycNET, Web of Science, Science Direct, Pub Med, SAGE Journals. Secondary search was carried out on Google Scholar, that also indexes preprint servers, such as psyArxiv. Each database was searched using a combination of search terms relating to the mental health disorder (anxiety, anxiety disorder, anxious, nervousness), population age (adolescent, youth, child, teenage, children, underage), intervention and related terms (therapy, treatment. intervention), type of intervention to focus the search on digital methods (technology-assisted, computerized, internet, smartphone, family-focused, parent-based, ICBT, eCBT, application), study design (RCT or randomized controlled trial), and intervention type (CBT, cognitive behavioral). The exact search string can be found in the supplementary material. The reference lists of eligible studies were also screened to identify potential articles.

Six independent researchers conducted the systematic screening of studies based on title and abstract. Each article was screened by two independent individuals. In case of exclusion of a study, the reason for exclusion also had to be provided. In case of a disagreement regarding inclusion of a study the principal investigator made the final decision. After the removal of duplicates, and research papers with potentially overlapping samples, the full-text of the remaining hits were examined by two independent raters, and two researchers extracted data from the screened articles. The first search was carried out in 2019 December, and an additional search in July, 2021.

### Risk of Bias

All included studies were evaluated for risk of bias by two raters, using the updated version of the Cochrane risk-of-bias tool (RoB 2, Sterne et al., 2019). This tool includes biases arising at different domains, based on both empirical evidence and theoretical considerations.

Studies were rated on the following factors with the use of signaling questions. (1) Randomization process. (2) Deviation from intended interventions. (3) Missing outcome data (e.g. evidence that the result was not biased by missing outcome data,). (4) Measurement of the outcome (e.g. whether the method of measuring the outcome was appropriate, blinding of outcome assessors). (5) Selection of the reported results (e.g. pre-specified analysis plan; whether the numerical result is likely to have been selected from multiple eligible outcome measurements). The risk for each bias was rated in three categories: high, low, or some concerns for each study. The algorithm provides an automatic rating based on the entered characteristics of the study, and the assessor also provides a rating. The judgements within each domain lead to an overall judgement, both by the tool itself and by the assessor. In case of divergent ratings, a consensus was reached after discussion between the two raters, re-examining signaling questions. Studies characterized by a low risk of bias were rated high quality, those with a high risk of bias low quality, and those with a „some concerns” rating were left labeled as „some concerns”. Moderation analysis was carried out considering different levels of risk of bias.

### Data Extraction

Two independent researchers coded each included study regarding study characteristics (study title, authors, year of publication, country, study quality), treatment characteristics (therapist involvement, family- or youth focus, type of intervention, length of intervention, place of delivery, number of completed sessions), sample characteristics (sample size, mean age, female ratio, clinical status) and outcome type (clinical severity ratings or self-report ratings of children; anxiety measure). As only few studies reported follow-up measures, anxiety levels at post-intervention were used.

In our study, we utilized a classification system for digital interventions that focuses on the extent of therapist involvement and the degree of integration between digital components and traditional therapy. This system encompasses standalone, unguided, guided, and blended care interventions. Standalone interventions are self-contained programs that do not require any additional human support or interaction, while unguided interventions may include some level of monitoring or minimal human support, like automated reminders or feedback. Guided interventions involve more significant human support, usually from a mental health professional or a trained coach, who provide personalized feedback, motivation, and guidance to users. Lastly, blended care interventions combine traditional face-to-face therapy with digital components, allowing clients to receive both in-person and online support from mental health professionals (Andersson & Cuijpers, 2009; Baumeister et al., 2014; Erbe et al., 2017; Karyotaki et al., 2017).

The categorization of family- or youth focused were defined as the following based on MacDonell & Prinz (MacDonell & Prinz, 2017) studies were labeled youth-focused, if they did not actively engage either of the parents or caretaker (no other tasks for parents other than giving consent, participating in assessment, and assisting in making the intervention accessible). Those which actively engaged one of the parents (e.g. session or self-help material delivered to parents regarding how to help their anxious child) were labeled as family-focused.

### Data Analyses

Pooled effect sizes were calculated in R 4.0.2 (Team, 2020) using the metafor 2.4.0 (Viechtbauer, 2010) and visualizations were created using the metaviz 0.3.1 package (Kossmeier et al., 2020). In order to calculate effect sizes, we extracted different sources of data depending on which was available in the study. Where possible, post-intervention mean, standard deviation and sample size of the intervention and control group was involved. In other cases test statistics or p-values and sample sizes were used. Hedges’ g of individual studies and overall effect size is reported, values from 0.2 signifying a small effect, those from 0.5 a medium effect, and around 0.8 or higher a large effect (Borenstein et al., 2009). Pooled effect sizes were calculated using a random-effects model with REML estimation. To calculate the heterogeneity of effect sizes the Q statistic and *I*^2^ statistic is used. We also conducted pre-specified moderation analyses to investigate the influence of family or youth focus, therapist involvement, place of delivery and study quality on treatment efficacy. An assessment of female ratio, drop-out rate and number of completed modules was also carried out, although not stated in our preregistration plan. Publication bias was assessed through visual inspection of the funnel plots (Sterne et al., 2011), the trim-and fill procedure (Duval & Tweedie, 2000), Egger’s test (Egger et al., 1997).

## Results

### Characteristics of Included Studies

**Fig. 1:**
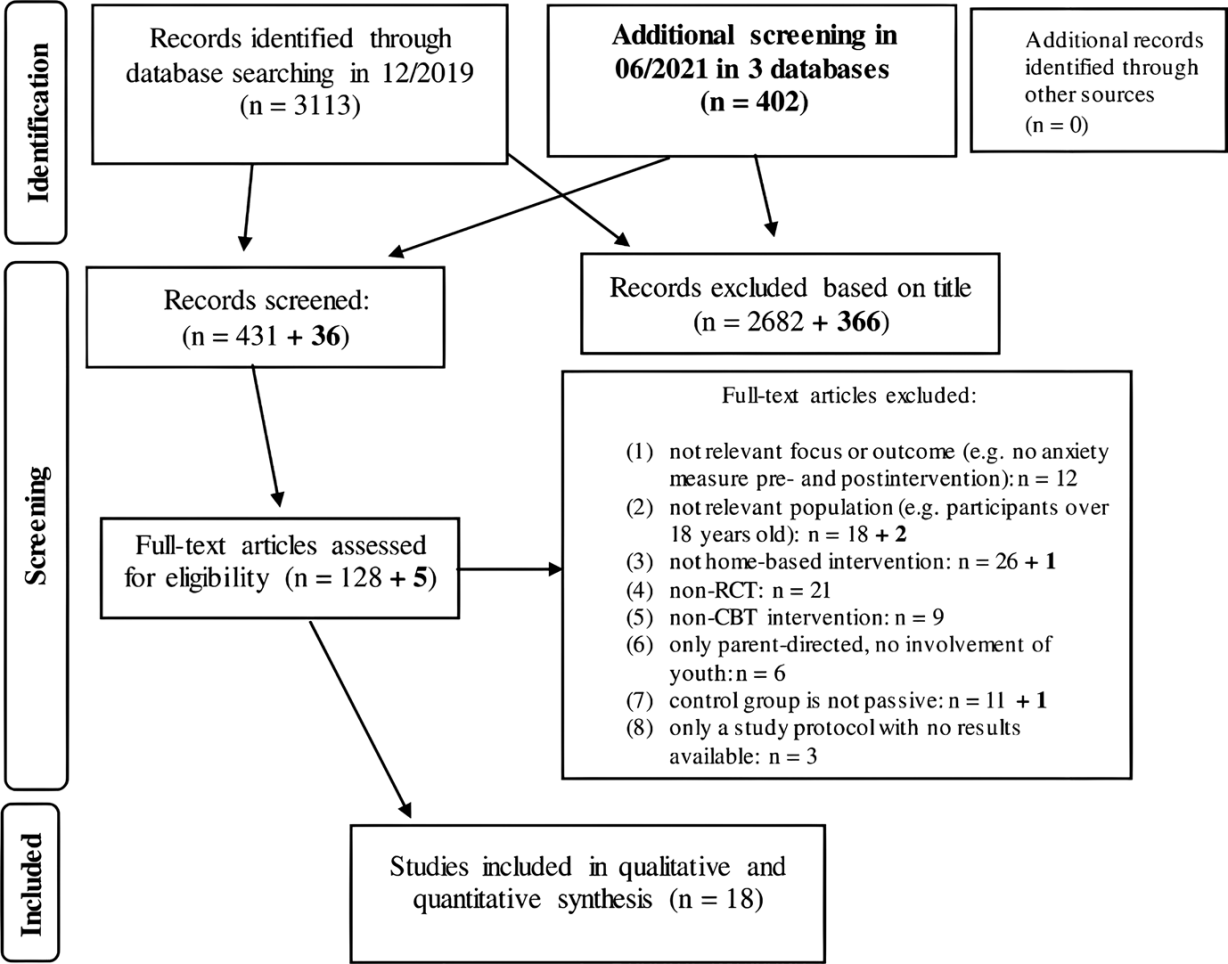
Systematic search flow of study. Second search is indicated in bold

The first systematic search in December, 2019 yielded 3113 results in the primary databases. After screening article titles, 431 articles were selected, out of the 431 articles 128 full-text articles were screened for inclusion and exclusion criteria. With removing duplicates, studies with potentially overlapping samples, and those not fully fulfilling inclusion criteria, 17 studies were included and coded in the final database. Most studies were excluded for lack of randomization and lack of passive (waitlist) control group. An additional second search was carried out in July, 2021 in order to account for recent publications (2020–2021), with only one study fitting our inclusion–exclusion criteria from this time frame. See detailed flow of systematic search on Fig. 1.

#### Study Characteristics

Altogether, the 18 included studies examined 1290 underaged individuals, with sample sizes ranging between 24 to 130. Only studies including children aged 18 or younger were analyzed in our study, and about half (N = 10) included children (12 years or below), and the rest (N = 8) adolescents (13–18 years). The overall mean age is 12.07 (SD = 2.85). Most studies were conducted in Australia (N = 10), others in the United Kingdom (N = 4), Sweden (N = 2), Canada (N = 1) or New Zealand (N = 1). All comparison groups included were waitlist control groups, in which participants began treatment after post-intervention measurements of the original intervention sample.

#### Mental Health Problem Characteristics

All interventions targeted reducing anxiety disorders with two exceptions (Fleming et al., 2012; Smith et al., 2015). The mentioned exceptions primarily targeted depression, but had a population with elevated levels of anxiety. In most cases, the examined population were participants with clinical levels of an anxiety-related mental disorder (different anxiety disorders: N = 11; mood disorder: N = 3; autism: N = 1; OCD: N = 1) and two studies conducted interventions on individuals without a clinical diagnosis.

#### Intervention Characteristics

Interventions were mostly low-intensity interventions, delivering one module per week, ranging from 5 to 16 modules. Two studies were standalone interventions, in two experiments participants received some level of monitoring, classifying as unguided programs, most studies (N = 13) were guided with a level of personalized therapist support and one included face-to-face sessions paired with online intervention. Five studies were solely youth-focused, and each of these had adolescents as participants, meanwhile thirteen actively engaged parents (family-focused) and examined children as well. Basic intervention, study and sample characteristics, and specifications on therapist involvement are presented in Table 1.

**Table 1:** Basic characteristics of included studies in the metaanalysis. Risk of bias was evaluated using the Cochrane Collaboration criteria and categorized into three groups: High, Low, or Some Concerns

Cobham (2012)	**Sample:** Anxiety disorder	**Intervention:** Family-focused, 12 sessions
	**Total N** = 32	**Method:** Guided (fortnightly telephone contact with therapist, which in average lasted 12 min)
	**Post-intervention N** = 32	**Bibliotherapy**: A family-focused approach where parent and child work through 12 sessions of a parent and child program using workbooks
	**M age** = 10	**Elements of CBT included**: Psychoeducation, coping strategies, cognitive restructuring, avoidance management, parent-assisted exposure tasks
	**Female %** = 47%	**Risk of bias:** Low
		**Control group**: Waitlist
		**Delivery setting**: Home
Conaughton et al. (2017)	**Sample:** Autism spectrum disorder	**Intervention:** Family-focused, 16 sessions
	**Total N** = 42	**Method:** Guided (weekly online contact with therapist in response to session activities + one phone call midway through the program)
	**Post-intervention N** = 42	**BRAVE-ONLINE**: Developed to treat anxiety disorders with 10 child and 6 parent sessions delivered weekly via the Internet
	**M age** = 10	**Elements of CBT included:** Psychoeducation, relaxation strategies, cognitive restructuring, graded exposure, contingency management techniques
	**Female %** = 14%	**Risk of bias:** Low
		**Control group:** Waitlist
		**Delivery setting:** Home
O’Connor et al. (2020)	**Sample:** non-clinical with elevated anxiety symptoms	**Intervention:** Youth-focused, 8 sessions
	**Total N** = 70	**Method:** Unguided
	**Post-intervention N** = 38	**BREATHE**: A newly developed online CBT program including multimedia-based education and activities that teach anxiety management through 8 modules to adolescents
	**M age** = 15	**Elements of CBT included**: Psychoeducation, cognitive distortions, relaxation skills, avoidance management, constructing a fear hierarchy
	**Female %** = 90%	**Risk of bias:** High
		**Control group**: Website
		**Delivery setting:** Home
Fleming et al. (2012)	**Sample:** Mood disorder	**Intervention:** Youth-focused, 7 sessions
	**Total N** = 32	**Method:** Guided (weekly contact with mental health professional to address any concerns or to provide support with the program)
	**Post-intervention N** = 30	**SPARX**: A 7-module program that takes place in a game world, where individuals inhabit a personalized character and help restore the balance in the game world by using skills from a “shield against depression”
	**M age** = 15	**Elements of CBT included:** Psychoeducation, relaxation skills, problem solving, activity scheduling, challenging and replacing negative thinking and social skills
	**Female %** = 44%	**Risk of bias:** Low
		**Control group:** Waitlist
		**Delivery setting:** School
Infantino et al. (2016)	**Sample:** Anxiety disorder	**Intervention:** Family-focused, 10 sessions
	**Total N** = 24	**Method:** Unguided
	**Post-intervention N** = 24	**Turnaround:** An audio intervention designed for 6–12 year old children with anxiety disorders. Contains 10 lessons, daily journal exercises, relaxation CD and two parent CDs
	**M age** = 7.5	**Elements of CBT included**: Psychoeducation, relaxation skills, cognitive restructuring, problem solving, exposure, contingeny management
	**Female %** = 54%	**Risk of bias:** Low
		**Control group:** Waitlist
		**Delivery setting:** Clinic
Lenhard et al. (2017)	**Sample:** OCD	**Intervention:** Family-focused, 12 sessions
	**Total N** = 67	**Method:** Guided
	**Post-intervention N** = 67	(patients have regular asychnronous contact with clinician through messages and occasionally via telephone; in average 17 min per week)
	**M age** = 15	**BiP OCD:** Designed for adolescents with OCD, consists of 12 modules with texts, animations and films and exercises for adolescents to do on their own and together with parents
	**Female %** = 46%	**Elements of CBT included: P**sychoeducation, cognitive strategies, exposure and response prevention, problem solving, relapse prevention
		**Risk of bias:** Low
		**Control group:** Waitlist
		**Delivery setting:** Home
Lyneham and Rapee (2006)	**Sample:** Anxiety Disorder	**Intervention:** Family-focused, 12 sessions
	**Total N** = 43 (email), 50 (phone)	**Method:** Guided
	**Post-intervention N** = 40 (email), 50 (phone)	(telephone or email-contact groups, in average 9 calls of 20 min and 21 emails in the other group)
	**M age** = 9	**Bibliotherapy:** A parent-implemented program adapted from the standard group CBT treatment. 12 modules for parents and children, using workbooks and a self-help book
	**Female %** = 49% (email), 48 (phone)	**Elements of CBT included:** Same as in Cobham (2012)
		**Risk of bias:** Some concerns (ADIS conducted with parents unblind to treatment condition, pre-specified analysis plan)
		**Control group:** Waitlist
		**Delivery setting:** Home
March et al. (2008)	**Sample:** Anxiety Disorder	**Intervention:** Family-focused, 16 sessions
	**Total N** = 73	**Method:** Guided (as mentioned above)
	**Post-intervention N** = 59	**BRAVE-ONLINE:** (as mentioned above)
	**M age** = 10	**Elements of CBT included:** (as mentioned above)
	**Female % = **55%	**Risk of bias:** Low
		**Control group:** Waitlist
		**Delivery setting:** Home
McLoone and Rapee (2012)	**Sample:** Non-clinical	**Intervention:** Family-focused, 10 sessions
	**Total N** = 87	**Method:** Guided (two group information sessions which helped with program delivery)
	**Post-intervention N** = 63	**Cool Kids at home**: A program implemented by parents with 10 modules, a leaders’ manual, a self-help book for parents and a workbook for children
	**M age** = 9	**Elements of CBT included: P**sychoeducation, cognitive distortions, exposure, social skills
	**Female % = **61%	**Risk of bias:** High: baseline differences suggest a problem with the randomization process, no evidence that result was not biased by missing outcome data, no pre-registered plan
		**Control group:** Waitlist
		**Delivery setting:** Home
Smith et al. (2015)	**Sample:** Mood disorder	**Intervention:** Youth-focused, 8 sessions
	**Total N** = 112	**Method:** Standalone
	**Post-intervention**	**Stressbusters**: A computerized-CBT program designed specifically for adolescents with depression, consists of 8 modules
	**N** = 110	**Elements of CBT included**: Psychoeducation, behavioural activation, cognitive restructuring, problem solving, social skills, relapse prevention
	**M age** = 14	**Risk of bias:** Low
	**Female %** = N/A	**Control group:** Waitlist
		**Delivery setting:** School
Spence et al. (2011)	**Sample:** Anxiety disorder	**Intervention:** Family-focused, 16 sessions
	**Total N = **71	**Method:** Guided** (**as mentioned above)
	**Post-intervention N = **65	**BRAVE-ONLINE:** as mentioned above
	**M age = **14 **Female % = **59%	**Elements of CBT included**: as mentioned above
		**Risk of bias:** Low
		**Control group:** Waitlist
		**Delivery setting**: Home
Spence et al. (2017)	**Sample:** Anxiety disorder	**Intervention:** Family-focused, 16 sessions
	**Total N = **125 **Post-intervention N = **98	**Method:** Guided (as mentioned above)
	**M age = **11 **Female % = **60%	**BRAVE-ONLINE:** as mentioned above
		**Elements of CBT included**: as mentioned above
		**Risk of bias:** Low
		**Control group:** Waitlist
		**Delivery setting:** Home
Stjerneklar et al. (2019)	**Sample:** Anxiety disorder	**Intervention:** Youth-focused, 8 sessions
	**Total N = **70	**Method:** Guided (weekly phone call from therapist set to a duration of approximately 20 min)
	**Post-intervention N = **70	**ChilledOut:** An online treatment program based on the Cool Kids and Chilled anxiety management programs. Teaches CBT strategies to adolescents
	**M age = **15 **Female % = **79%	**Elements of CBT included:** Psychoeducation, cognitive restructuring, fear hierarchy, problem solving, relaxation skills, assertive communication, relapse prevention
		**Risk of bias:** Low
		**Control group:** Waitlist
		**Delivery setting:** Clinic
Thirlwall et al. (2013)	**Sample:** Anxiety disorder	**Intervention:** Family-focused, 8 sessions
	**Total N = **130	**Method:** Blended (“Brief guidance”: fortnightly therapist contact; two 1-h face-to face sessions and two telephone sessions)
	**Post-intervention N = **109	**Parent-delivered CBT:** 8 modules with an additional self-help book and homework for parents independently and together with their child
	**M age = **10	**Elements of CBT included:** Psychoeducation, cognitive restructuring, exposure plan, problem solving
	**Female % = **49%	**Risk of bias:** Some concerns (no blinding of clinicians, no pre-specified analysis plan)
		**Control group:** Waitlist
		**Delivery setting:** Home
Vigerland et al. (2016a, 2016b)	**Sample:** Anxiety disorder	**Intervention:** Family-focused, 11 sessions
	**Total N = **93	**Method:** Guided (online contact with therapist in written messages + three phone calls)
	**Post-intervention N = **90	**A CBT intervention focusing mainly on exposure:** Developed by research group, fit to primary diagnosis, with 7 modules aimed at parents and 4 at children
	**M age = **10	**Elements of CBT included:** Psychoeducation, coping strategies, problem solving, exposure
	**Female % = **55%	**Risk of bias:** Some concerns (no blinding of clinicians, no pre-specified analysis plan)
		**Control group:** Waitlist
		**Delivery setting:** Home
Waite et al. (2019)	**Sample:** Anxiety disorder	**Intervention:** Family-focused, 16 sessions
	**Total N = **60	**Method:** Guided (as mentioned above)
	**Post-intervention N = **48	**BRAVE-ONLINE:** as mentioned above
	**M age = **15	**Elements of CBT included**: as mentioned above
	**Female % = **65%	**Risk of bias:** Low
		**Control group:** Waitlist
		**Delivery setting:** Home
Wright et al. (2016)	**Sample:** Mood disorder	**Intervention:** Youth-focused, 8 sessions
	**Total N = **91	**Method:** Standalone (as mentioned above)
	**Post-intervention N = **83	**Stressbusters:** as mentioned above
	**M age = **16	**Elements of CBT included:** as mentioned above
	**Female % = **66%	**Risk of bias:** High (baseline differences and no adjustment for these differences, no pre-specified analysis plan)
		**Control group:** Placebo
		**Delivery setting:** Home
Wuthrich et al. (2012)	**Sample:** Anxiety disorder	**Intervention:** Family-focused, 8 sessions
	**Total N = **43	**Method:** Guided (8 phone calls between adolescent and therapist, and 3 phone calls between parent and therapist)
	**Post-intervention N = **37	**CoolTeens:** A computerized program based on the Cool Kids, teaches CBT skills to manage anxiety in 8 modules
	**M age = **15	**Elements of CBT included**: Psychoeducation, cognitive restructuring, graded exposure, goal setting, relapse prevention **Risk of bias:** Some concerns (no blinding of clinicians, no pre-specified analysis plan)
	**Female % = **63%	**Control group:** Waitlist
		**Delivery setting:** Home

#### Measures of Anxiety

##### Self-Report

We analysed the results of self-reported post-intervention anxiety measures of children and compared them with the results of those of the waitlist groups. Severity of anxiety symptoms were reported on the Spence Children’s Anxiety Scale (Spence, 1998) in 14 studies. The Revised Children’s Manifest Anxiety Scale (RCMAS, Reynolds & Richmond, 1997) was assessed in two studies, and Screen for Child Anxiety Related Disorders in one study (SCARED, Boyd et al., 2003) and the Multidimensional Anxiety Scale for Children–Second Edition (MASC2, March et al., 1997).

##### Clinician Rating

Where possible (N = 13) the diagnostic ratings of clinicians were also coded. Diagnostic status was assessed using the parent and child version of Anxiety Disorders Interview Schedule for DSM-IV, which reports sound psychometric properties (ADIS C/P, Silverman & Albano, 1996). A clinician severity rating (CSR) was assigned by the assessor based on the ADIS C/P, with a severity rating of 4 (moderate interference) indicating clinical significance. If a study included severity ratings of different diagnoses, the severity ratings – and therefore loss or ongoing presence – of the primary anxiety diagnosis was used. Out of the 13 studies, in 9 studies assessors were blind to experimental condition.

### Pooled Effect Sizes and Heterogeneity of Results

In order to investigate the efficacy of digital cognitive behavioral interventions compared to waitlist control groups, we ran random-effects analyses using restricted maximum likelihood estimation on data derived from 18 studies (children self-report ratings) and 13 studies (clinician ratings). Before calculating the Pooled effect sizes, we investigated if there are any outlier studies that should be excluded (Harrer, M. Cuijpers et al., 2019). We found one outlier study for the self-report outcome (Cobham, 2012; Infantino et al., 2016) and one outlier study for the clinician rating outcome (Cobham, 2012). These outlier studies reported much larger effects than the rest of the studies, therefore excluding them made the estimation of the pooled effect more conservative. We conducted all subsequent analyses without these studies.

**Fig. 2:**
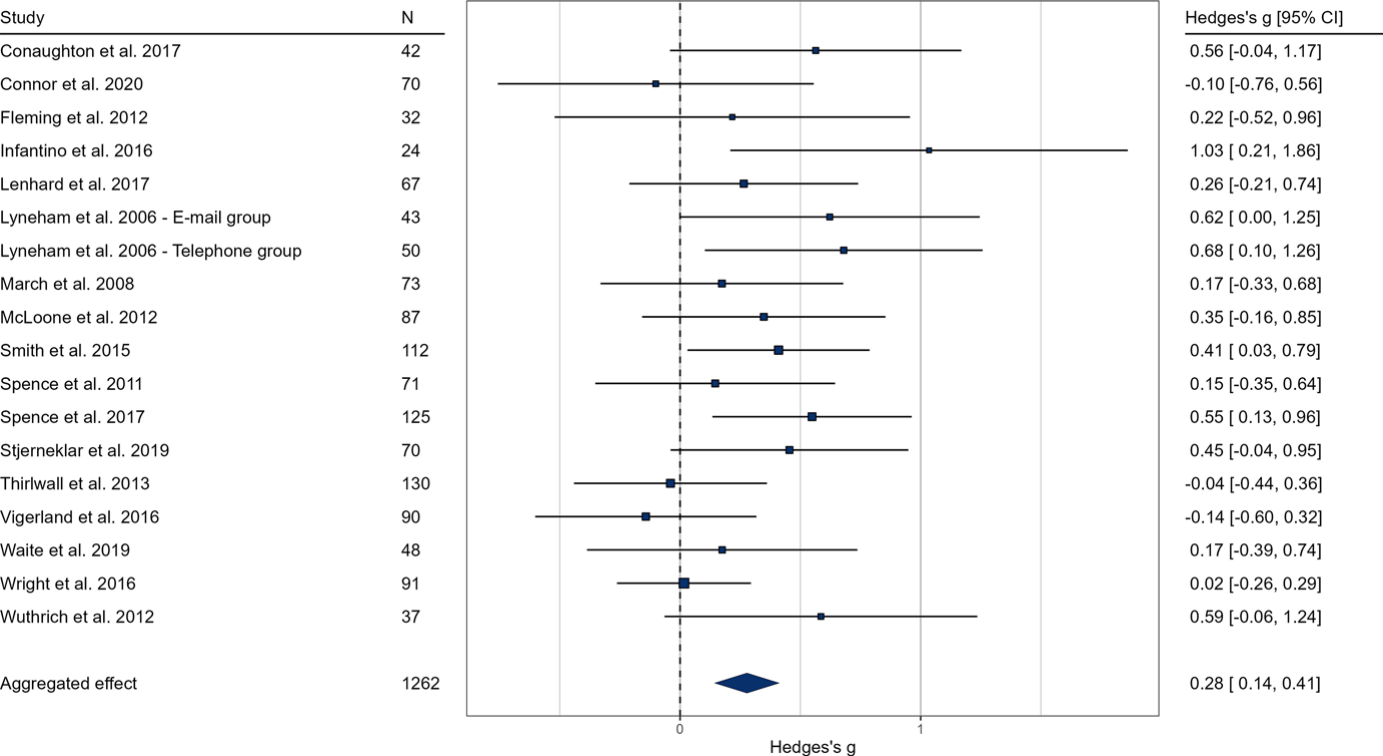
Forest plot of pooled effect sizes based on self-report anxiety ratings of children. In the results, values above 0 favor the intervention group. The lower and upper limits represent the 95% confidence interval boundaries for each study. The overall effect size is reported in the bottom row, with the filled diamond representing the average effect

**Fig. 3:**
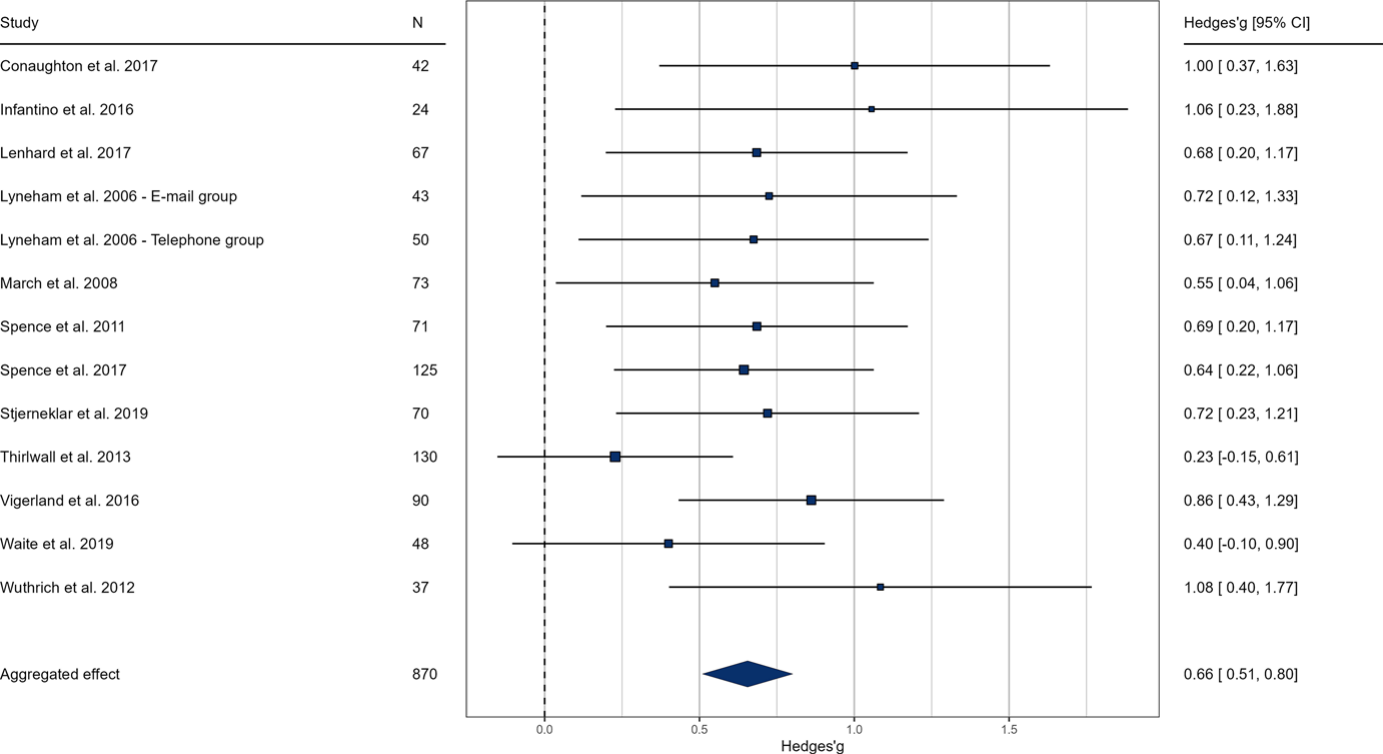
Forest plot of pooled effect sizes based on clinician severity ratings. In the results, values above 0 favor the intervention group. The lower and upper limits represent the 95% confidence interval boundaries for each study. The overall effect size is reported in the bottom row, with the filled diamond representing the average effect

**Fig. 4:**
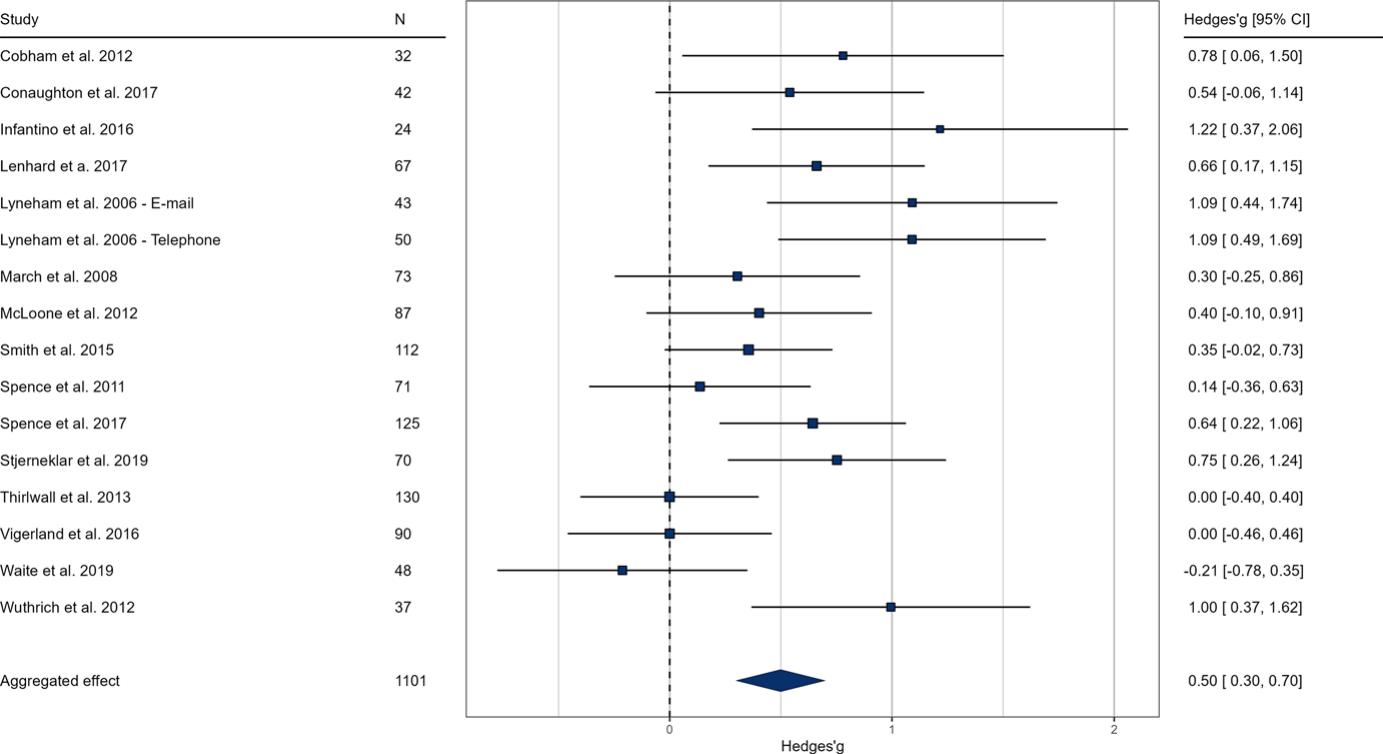
Forest plot of pooled effect sizes based on parental rating. In the results, values above 0 favor the intervention group. The lower and upper limits represent the 95% confidence interval boundaries for each study. The overall effect size is reported in the bottom row, with the filled diamond representing the average effect

Based on self-report ratings at post-intervention, the pooled effect size indicated a significant, but small difference in favor of the intervention groups compared to the control groups (g = 0.28, k = 18, *p* < 0.001, 95% CI [0.14; 0.41], see Fig. 2.). The anxiety-related severity ratings of clinicians at post-intervention indicated a medium sized effect of online interventions (g = 0.66, k = 13, *p* < 0.001, 95% CI [0.50; 0.80], see Fig. 3.). We examined parental ratings of anxiety which also indicated a medium effect size (g = 0.49, k = 16, *p* < 0.001, 95% CI [0.29; 0.69], see Fig. 4.) These results suggest that online interventions effectively reduce anxiety symptoms in children and adolescents, supporting our hypothesis. Moreover, our findings indicate that professionals and caretakers perceive these effects more prominently than the patients themselves.Results of heterogeneity analysis for both self-report and clinician ratings suggest a homogeneity in effect sizes; self-report: Q (df = 17) = 21.43, *p* = 0.207, I^2^ = 22.53%; clinician rating: Q (df = 12) = 10.60, *p* = 0.563, I^2^ = 5.47%).

### Moderation Analyses of Treatment Efficacy

Although heterogeneity of the studies did not prove significant in either of the outcomes, we carried out the pre-registered moderation analyses. As none of the investigated predictors yielded a significant result, we only report the omnibus tests for all variables instead of coefficients for all category levels.

Regarding self-reported anxiety, none of the following moderators were significant moderation: the effect of intervention focus (family or individual or mixed) QM(df = 3) = 1.55, *p* = 0.670; place of delivery (home or clinic or school) QM(df = 3) = 3.53, *p* = 0.317; average number of sessions QM (df = 1) = 0.01, *p* = 0.929; study quality QM(df = 2) = 5.38, *p* = 0.068; anxiety measure QM(df = 3) = 1.35, *p* = 0.716; intended length QM(df = 1) = 0.93, *p* = 0.335; average age QM(df = 1) = 1.61, *p* = 0.204; proportion of females QM(df = 1) = 1.05, *p* = 0.304; and diagnosis QM(df = 5) = 2.17, *p* = 0.825.

Regarding clinician rating, moderation analyses were not significant for the following predictors: the effect of intervention focus QM(df = 2) = 0.98, *p* = 0.612; therapist involvement QM(df = 1) = 0.91, *p* = 0.339; place of delivery QM(df = 1) = 0.54, *p* = 0.463; average number of sessions QM(df = 1) = 0.809, *p* = 0.368; study quality QM(df = 1) = 0.180, *p* = 0.672; intended length QM(df = 1) = 0.02, *p* = 0.885; average age QM(df = 1) = 0.00, *p* = 0.987; proportion of females QM(df = 1) = 0.17, *p* = 0.678; diagnosis QM(df = 2) = 1.17, *p* = 0.557; and blinding of the rater QM(df = 1) = 0.07, *p* = 0.789.

**Fig. 5:**
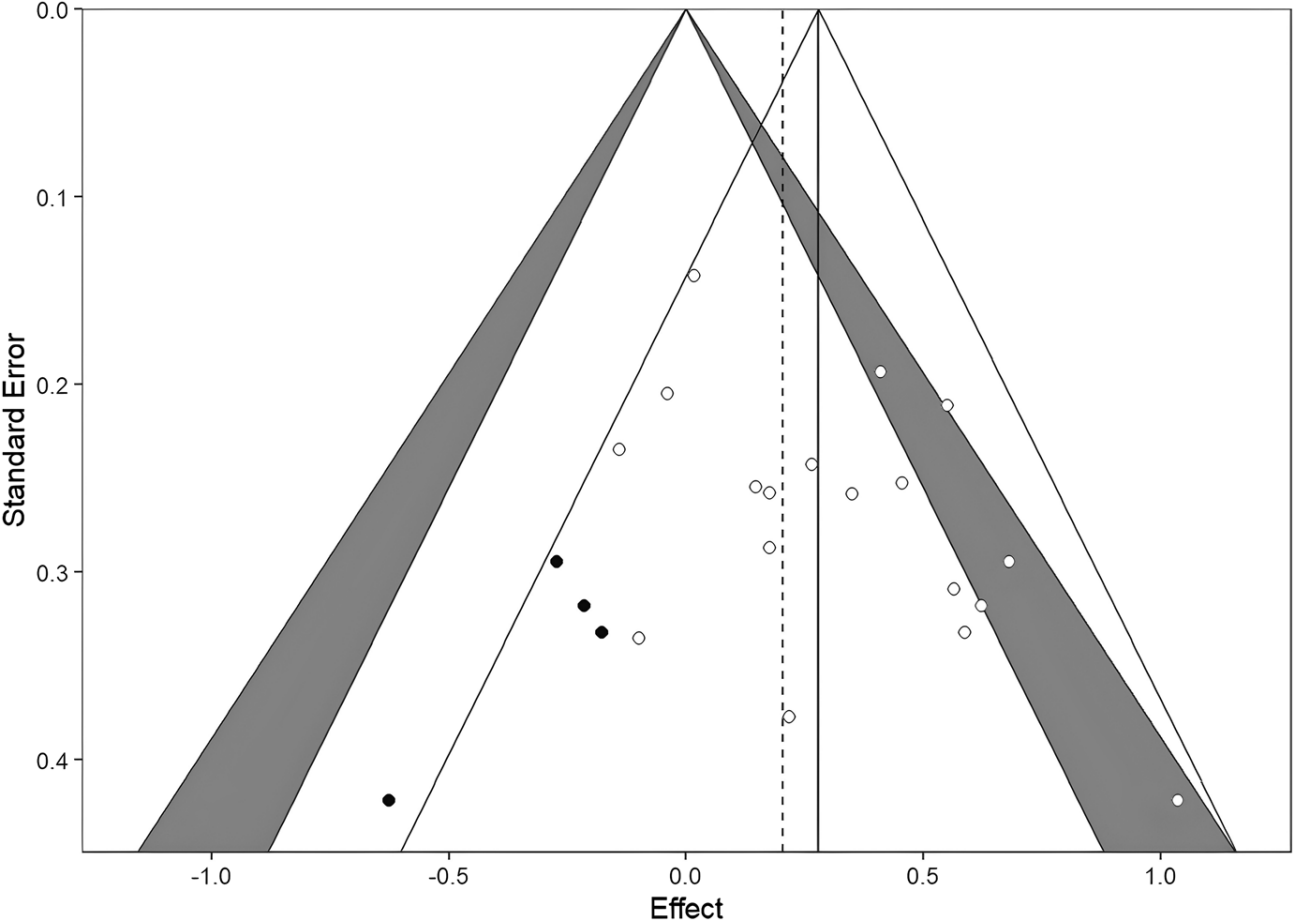
Contour-enhanced funnel plots for the self-reported outcomes, with trim and fill imputed studies. As models showed evidence of publication bias, we used the trim and fill method to impute effect sizes to correct the pooled effects. Self-reported, clinician-rated, and parent-rated models were corrected by 4, 4, and 3 effects, respectively (filled dots). All models remained significant after trim-and-fill imputed effects. Also, if we compare the overall effect (solid vertical lines) size with imputed effects (dashed vertical lines), the effects remained similar

**Fig. 6:**
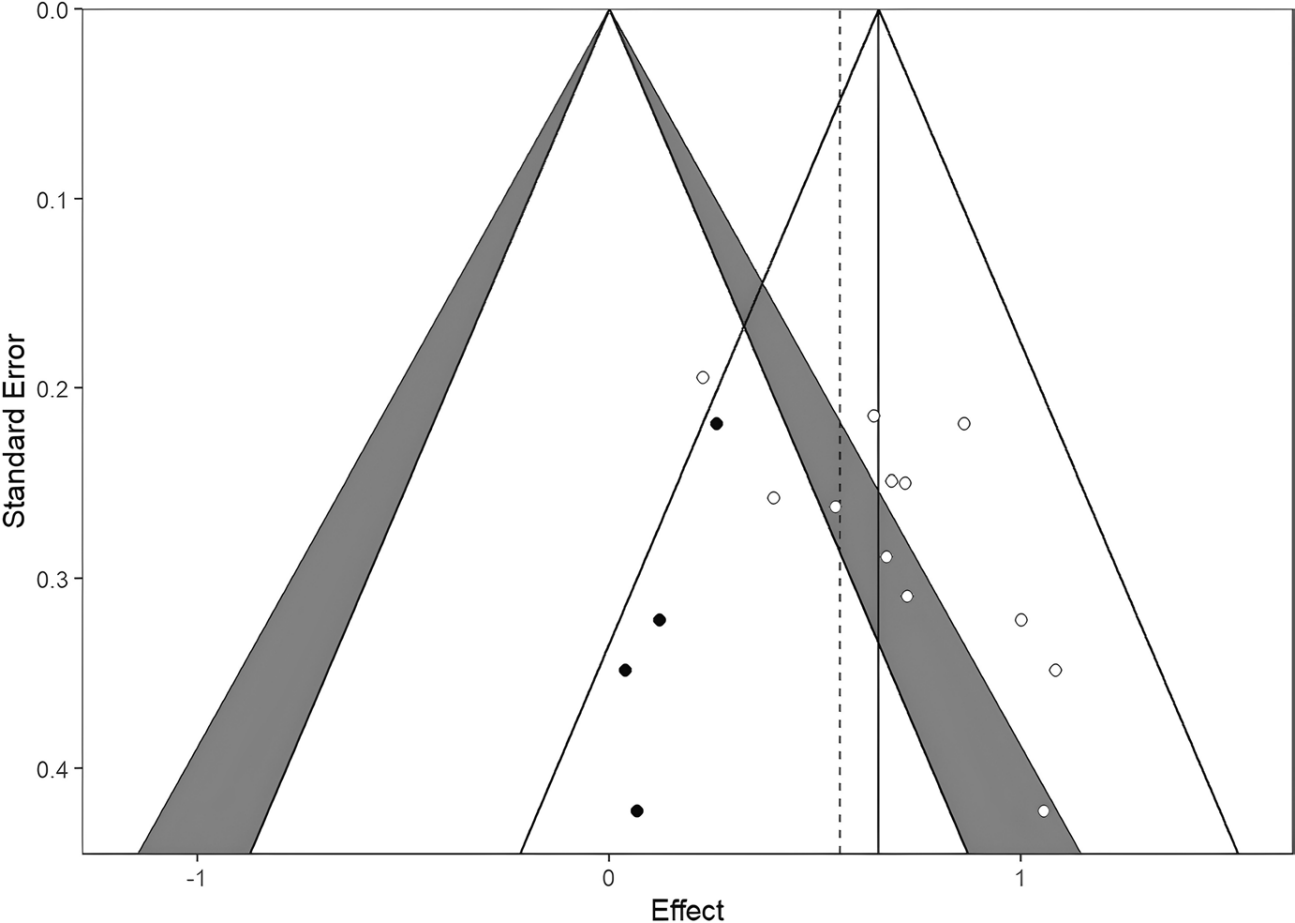
Contour-enhanced funnel plots for the clinician rated outcomes, with trim and fill imputed studies. As models showed evidence of publication bias, we used the trim and fill method to impute effect sizes to correct the pooled effects. Self-reported, clinician-rated, and parent-rated models were corrected by 4, 4, and 3 effects, respectively (filled dots). All models remained significant after trim-and-fill imputed effects. Also, if we compare the overall effect (solid vertical lines) size with imputed effects (dashed vertical lines), the effects remained similar

**Fig. 7:**
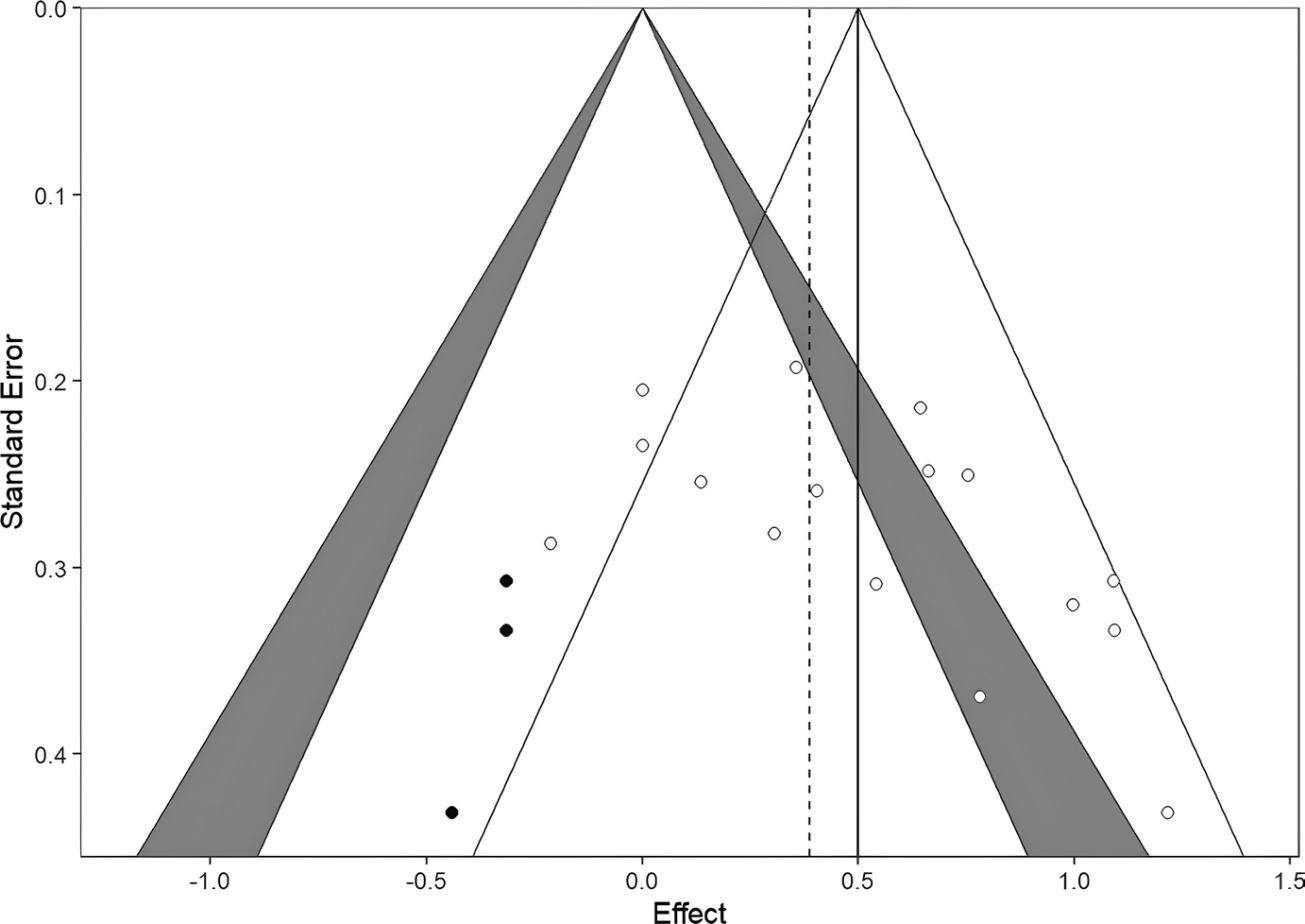
Contour-enhanced funnel plots for the parent rated outcomes, with trim and fill imputed studies. As models showed evidence of publication bias, we used the trim and fill method to impute effect sizes to correct the pooled effects. Self-reported, clinician-rated, and parent-rated models were corrected by 4, 4, and 3 effects, respectively (filled dots). All models remained significant after trim-and-fill imputed effects. Also, if we compare the overall effect (solid vertical lines) size with imputed effects (dashed vertical lines), the effects remained similar

Moderator analyses were also carried out on parental ratings, and used method was a significant predictor: QM(df = 10) = 19.34, *p* = 0.036. Other variables were not significant: intervention focus QM(df = 2) = 3.21, *p* = 0.200; therapist involvement QM(df = 2) = 0.41, *p* = 0.815; clinical status QM(df = 4) = 0.29, *p* = 0.990; place of delivery QM(df = 2) = 1.99, *p* = 0.368; intended length QM(df = 1) = 0.46, *p* = 0.497; study quality QM(df = 2) = 1.45, *p* = 0.484; average length QM(df = 1) = 0.46, *p* = 0.497; age QM(df = 1) = 0.58, *p* = 0.445; female ratio QM(df = 1) = 0.08, *p* = 0.769. (Figs. 5, 6, 7)

### Publication Bias

Publication bias analyses were carried out using funnel plots, Duval’s trim-and-fill procedure, and Egger’s regression separately on the three outcome measures. As discussed, effect sizes may indicate some publication bias, as the funnel plots show asymmetry (funnel plots are available on the link https://osf.io/yvdzs/files/osfstorage). Moreover, the trim-and-fill method imputed four studies for self-report, four studies for clinician rating and 3 studies for parental report in order to adjust for the potential publication bias. All models remained significant after trim-and-fill imputed studies, and the overall effect sizes remained similar with tram-and-fill studies included.

As for self-report ratings, Egger’s regression indicated asymmetry: t (16) = 2.27, *p* = 0.037 and the trim-and-fill adjusted effect size was was 0.20 CI 95% [0.07, 0.34], indicating a slight decrease in effect.

The funnel plot based on ratings of clinicians also showed asymmetry. The trim-and-fill method imputed four studies and the Egger’s regression also indicated asymmetry; t (11) = 2.90, *p* = 0.014. The trim-and-fill adjusted effect size was 0.56 CI 95% [0.42, 0.70], indicating a decrease in the effect that remained of medium magnitude.

On parental ratings, the funnel plot showed asymmetry. The trim-and-fill method imputed three studies and the Egger’s regression also indicated asymmetry; t (14) = 2.42, *p* = 0.029. The trim-and-fill adjusted effect size was 0.39 CI 95% [0.18, 0.61], indicating a slight decrease in effect. Overall, although funnel plots showed some asymmetry, it is unlikely that the pooled effect is heavily biased.

## Discussion

Our results suggest that anxiety may be effectively reduced in children and adolescents through digital interventions. This was observable on both self-reported levels of anxiety and ratings of their parents and clinicians as well. We found an overall small but significant effect in self-reported anxiety ratings of children after intervention, and a medium sized effect based on the ratings of clinicians and parents. The clinicians assessing post-intervention effects were in most studies blind to whether an individual received intervention or not, which makes the results even more promising. In many cases, children were free from their primary anxiety diagnosis after a digital intervention (e.g. Conaughton et al., 2017; Infantino et al., 2016; Vigerland et al., 2016a, 2016b). These findings are in line with and lend further support to previous evidence, that indicates the benefits of technology delivered CBT programmes (Andrews et al., 2010). Treatment effects proved to be homogenous for all outcomes, with one exception. Used method was a significant predictor of treatment efficacy in parental ratings, but not in other variables. Neither the characteristics of the intervention (length, place of delivery: home or clinic or school, involvement of a therapist, focus of the treatment: family or individual) nor the characteristics of the patients (average age, female ratio, diagnosis) nor the characteristics of the studies (quality, anxiety questionnaire, blinding of the raters) moderated the effect size. Based on these, both shorter and longer interventions may be effective (5–16 modules), and these programs can be effectively delivered at home. Standalone, unguided and guided programs all showed to be effective. The involved studies were mostly of high quality, using reliable and valid instruments to measure anxiety and blinding of raters when possible. We found some indication of publication bias, however trim-and-fill adjusted effect sizes did not suggest a large deviation from the observed pooled effects.

The number of underage individuals suffering from anxiety disorders is high. Thus developing new ways to treat these pathologies is essential (Firth et al., 2017). Computerized technologies and the ever-expanding reach of the internet have made it possible for children and adolescents to access mental health interventions in a way that is less limited in location and other constraining factors. Digital programs are a promising public health approach for the treatment of anxiety amongst children and could also reduce the cost of treatment (Yap et al., 2018). Furthermore, a barrier to prevent an individual from receiving traditional, face-to-face treatment could appear at any time either on the individual (e.g. sickness) or societal (e.g. restrictions, pandemic) level. Therefore, digital methods could offer a stable alternative. Several other advantages could also be mentioned in favor of digital programs including addressing staff shortages, maintaining privacy, permitting self-pacing and flexible scheduling (MacDonell & Prinz, 2017). Another of these important factors is that digital programs potentially reduce the stigma of dealing with a mental illness, as this seems to be an important underlying factor in refusing to seek help. It is also possible that modern technologies are more appealing for young individuals, which makes this form of treatment more engaging for them. By providing digital, technology enhanced treatments, subpopulations that would be unreachable for traditional interventions may now have an opportunity for treatment.

Different issues, such as the main moderators of treatment efficacy were also addressed. According to our results the treatment effects were rather homogenous and only one of the examined moderators proved to significantly contribute to treatment efficacy. However, this current study could only investigate the characteristics of the interventions and the patients. Some contextual factors may still be important moderators of the efficacy of digital treatments. Grist et al. (2019) in their meta-analysis, found that parental and therapist involvement had an impact on technology delivered therapy outcomes. This is in contrast to the results of this present meta-analysis. This may be due to the fact that Gris et al. (2019) not exclusively examined CBT interventions, while this present meta-analysis only involved studies that examined CBT based interventions. Parental and therapist involvement may influence the results of different methods with a different impact. When methods not examined separately, it is difficult to draw method specific conclusions.

There is some evidence on family input in CBT for child anxiety disorders, showing that results highly differ from study to study as parents are involved in meaningfully different ways (Reuman et al., 2021). For example, parents often serve as a collaborator in treatment rather than a co-client, which does not consistently lead to superior results compared to individual CBT (Kendall et al., 2008). Parents who engage in’anxiety-enhancing’ parenting behaviours may be less likely to assist in the adaptive cognitive, social and emotional development of their children (Ginsburg & Schlossberg, 2002). Along the same vein, actively involved parents may boost the effectiveness of the intervention significantly by jointly working on managing anxiety or through changed family dynamics. This is also supported by the observation that the two studies in our meta-analysis with the largest effect sizes also actively engaged parents (Cobham, 2012; Infantino et al., 2016). In fact, these studies reported effect sizes that were identified as outliers, and therefore could not be compared directly to the other studies. These results highlight that a parent's role in the management of child anxiety may be important but not completely essential. Involving parents may help to change their reactions or parenting behaviors related to anxiety. Additionally, it is possible that by managing their anxiety on their own, children can feel more self-efficacy.

Regarding therapist involvement, we found that interventions with and without therapist contact were similarly effective in reducing anxiety based on the self-report of children. Unfortunately, due to the lack of studies we could not investigate this effect on the clinician ratings. It is possible that the involvement of a therapist is not just relevant for the treatment outcome, but also for increasing patient retention and adherence. Prior research have suggested minimal phone contact with therapists to encourage program completion (Newman et al., 2011). Newman and colleagues also suggest that the most efficacious level of therapist contact varies by disorder.

One of the limitations of self-directed programs identified by Cobham (2012), is the high dropout rate amongst technology-mediated anxiety interventions for children. As opposed to Cobham (2012) we did not find high average dropout rates in the studies we involved in this meta-analysis. According to our analysis the average dropout rate was 5.25% for self-report and 4.77% for clinician rating in the included studies in this meta-analysis.

Some aspects of digital programs could still be improved. For example, apart from therapeutic alliance, challenges of these interventions also arise from their dependency on technological tools. As many of the existing digital interventions are technology-assisted (Dèttore et al., 2015; Hall & Bierman, 2015; MacDonell & Prinz, 2017; Wuthrich et al., 2012) these are only available to those with regular personal access to technology and the internet. Although internet penetration rates have climbed rapidly during the past decades, there still exists a problem of “digital divide” (Gonzales, 2016; Wamuyu, 2017). In other words, families with low social-economic status may not be able to use these programs. Several studies reveal problems in low income neighborhoods, e.g. that many people struggle to maintain stable access and therefore have negative attitudes toward internet adoption (Gonzales, 2016). With the increased access to smart phones, future interventions may move toward smart phone based programmes instead of computerized ones, which might make this type of treatment more accessible for low-income families. However, the issue of internet data costs may still arise (Freeman et al., 2020).

An issue of confidentiality and data protection may also arise, as well as the unreliability of technological equipment.

### Usefulness of Digital Interventions

To evaluate the usefulness of digital cognitive behavioral interventions, we gathered information on treatment credibility, satisfaction, adherence, completion of modules, and obstacles to improvement reported in the studies.

The treatment expectancy and credibility ratings of both children and parents revealed a moderate to strong inclination towards positive outcomes of digital treatment interventions investigated, despite the limited availability of data from only three studies (Infantino et al., 2016; March et al., 2008; Spence et al., 2011).

A higher proportion of studies examined treatment satisfaction post-intervention. In all cases, authors report moderate to high levels of satisfaction (e.g. Cobham, 2012; Conaughton et al., 2017; Infantino et al., 2016; Lenhard et al., 2017; March et al., 2008; Spence et al., 2017; Vigerland et al., 2016a, 2016b). In the study of Lenhard el at (2017) the authors noted that half of the participants required additional face-to-face sessions alongside the internet-delivered program, even though only 4% of them would have preferred face-to-face treatment. According to the findings of Spence (2011), comparing a digital intervention to a traditional one as well, while parents generally expressed satisfaction with the online intervention, those who underwent face-to-face treatment exhibited slightly greater program satisfaction.

 A significant advantage of digital interventions could be their ability to allow for self-pacing and flexibility, although this could pose a challenge to adherence and to the comparison of data with offline therapies that adopt a more structured approach. Studies involved in our research often report that participants did not complete all modules by post-intervention, and tended to work their way more slowly through session than may be the case with clinic-delivery (e.g. March et al. (2011), Spence (2017), Vigerland). According to March et al. (2008), at posttreatment, only 60% of parents and 33.3% of children had completed all treatment sessions in their experiment. However, by 6-month follow-up,this percentage increased to 72.3% of parents and 62% of children. This could be a likely explanation for the delay in finding significant reductions in primary clinical diagnosis. Cobham (2012) also emphasizes a more flexible and individualized “therapy schedule”. as a possible explanation in low attrition rates, and adds therapist-initiated telephone contact as an important factor to improve adherence.

The completion of therapy sessions and an increase in the duration of practicing the acquired skills may be crucial in providing effective treatment. Nevertheless, the existing results are slightly contradictory regarding the precise nature of this association (Spence et al., 2017; Vigerland et al., 2016a, 2016b). Spence (2017) reported that a higher number of completed therapy sessions at six months (but not at 12 weeks) was significantly linked to greater clinical improvement based on clinical severity scales, although this was not observed for the self-report measures of parents and children. Furthermore, she deduced that a higher completion rate of sessions was associated with more substantial reductions in anxiety levels and enhanced functioning among children but not adolescents.

As previously stated, digital interventions can overcome various obstacles that impede individuals from seeking conventional psychological assistance. Nevertheless, these programs are not without their own barriers. McLoone (2012) mentions “treatment demands”, “perceived relevance”, and “stressors and obstacles that compete with treatment” as the most significant difficulties reported by parents in their study. In Wuthrich’s study (2012), participants listed the greatest barrier to treatment is “finding time”. These factors may be associated with the resources available to families, as some studies have noted that their findings may be constrained in terms of generalizability due to a high socioeconomic background and a high level of parental education. This may be because such families may be better equipped to assume the role of therapists for their child or provide a serene environment that allows for the thorough implementation of the program (Infantino). Conversely, McLoone (2012) performed analyses to compare whether there was an association between the conventional impediments to treatment, such as low parental education levels or single-parent family structures, and the number of sessions completed by the parent. None of the analyses demonstrated statistical significance, which enhances the plausibility of digital interventions being advantageous to individuals who might otherwise be unable to attend therapy.

It is important to acknowledge that while digital programs have demonstrated moderate to high levels of treatment credibility and satisfaction, and can surmount certain obstacles by offering flexibility and expanding treatment access to a broader population, they still appear to have limitations regarding the pathologies that can be effectively treated through their use.

For example, regarding social phobia, social anxiety disorder, or those with a high degree of comorbidity or severity, longer, more intensive treatments may be needed, including intensive, invivo exposure and practice of cognitive and behavioral skills (Spence et al., 2011, 2017; Vigerland et al., 2016a, 2016b). In such instances, supplementing digital programs with face-to-face therapy may be especially beneficial, and further investigations are warranted to determine the degree to which digital therapies can aid in the recovery from these disorders.

### Limitations

Due to the relatively low number of studies, it was not possible to compare the specific interventions. The type of technology used in the interventions was not examined as a moderating factor. The type of technology and graphics used may make an intervention more accepted amongst the youth and may even impact the dropout rate. This could be examined in future studies. Intervention characteristics were not examined in this meta-analysis. The analysis of specific characteristics (such as psychoeducation, relaxation training, recognition of the physiological symptoms of anxiety, cognitive strategies of coping self-talk and cognitive restructuring, graded exposure, and problem-solving) may provide a deeper understanding of what specific characteristics have an impact on the results.

On the other hand, as technologies are improving rapidly the interventions may become obsolete in a few years’ time. This might make the estimation of a general treatment effect more meaningful than looking for the effectiveness of specific digital therapies.

The included studies typically did not use long-term follow-up measurements. These would be crucial to estimate the longevity of the interventions. In order to adjust for the publication bias, further literature search could be extended to doctoral dissertations and RCT registries, and authors of related publications could be contacted. This way gray literature that is not indexed by the currently used databases could be included in the study. As previously mentioned, further research is needed to examine the effect of therapist involvement as well, and not only the contact time, but the manner of the contact may be of importance (e.g. telephone contact may be more efficient than email contact).

In our meta-analysis, we only included waitlist or other non-active methods as control group. Nevertheless, conducting studies that compare traditional methods of care to their digital counterparts could provide valuable insights into this field.

Dropout rate could be a moderating factor affecting the results of the meta-analysis. Future studies could involve dropout rate as a moderating factor in their analysis.

## Conclusion

According to the results, digital interventions proved to be effective for reducing anxiety in children and adolescents. Regardless of the existing limitations of digital programs, potential benefits may go far beyond obstacles, and could provide a new direction in anxiety treatment and prevention for the youth. Solely youth-focused interventions also showed to be efficacious in our meta-analysis, meaning that underage individuals have the possibility of improving their mental health independently. Further studies are needed to examine whether family-focused interventions may also result in further advantages whilst contributing to several factors of improved family dynamics and well-being. With the assistance of technology and parents, a growing number of children could be provided continuous treatment and be possibly saved from the detrimental effects of anxiety.

### Supplementary Information

Below is the link to the electronic supplementary material.

Supplementary file1 (DOCX 12 kb)
