# Supplementary material for: Cognitive Behavioral Digital Interventions are Effective in Reducing Anxiety in Children and Adolescents: A Systematic Review and Meta-analysis
Source: J Prev (2022). 2023 Dec 14;45(2):237–67. doi: 10.1007/s10935-023-00760-0 (PMC10981643; doi:10.1007/s10935-023-00760-0)
Supplement: Supplementary file 1 — Supplementary file1 (DOCX 12 kb) [file 10935_2023_760_MOESM1_ESM.docx]

# Supplementary material

Appendix A: Search String by Databases
Systematic search was done in two flows: in December, 2019, and July, 2021.

**PsycNET (87 results), PubMed (299 results), Web of Science (861)**

(adolescent or yout* or children or underage* or child or teen*)

AND (anxiety* or anxious*)

AND (CBT* or cog* behav*)

AND (random* cont* trial* or RCT or random*)

AND (online or web or techn* or parent* or computer*)

AND (interven* or therap* or progr*)

**Science Direct** (1003 results)

(adolescent OR children ) AND "intervention" AND "anxiety" AND (CBT OR cogn* behav*) AND (RCT OR randomized controlled) AND (internet-based OR parent-guided OR technology-mediated OR computerized OR self-guided OR technology-assisted OR technology-based OR self-help)

**SAGE journals** (863 results)

(adolescent OR children ) AND "intervention" AND "anxiety" AND "CBT" AND (RCT OR randomized controlled) AND (internet-based OR parent-guided OR technology-mediated OR computerized OR self-guided OR technology-assisted OR technology-based OR self-help)

Appendix B: Open access database

https://osf.io/bqy2p/
